# Crystal structure of the membrane (M) protein from a bat betacoronavirus

**DOI:** 10.1093/pnasnexus/pgad021

**Published:** 2023-01-30

**Authors:** Xiaodong Wang, Yuwei Yang, Ziyi Sun, Xiaoming Zhou

**Affiliations:** State Key Laboratory of Biotherapy, Department of Integrated Traditional Chinese and Western Medicine, Rare Diseases Center, West China Hospital, Sichuan University, Chengdu, Sichuan 610041, China; State Key Laboratory of Biotherapy, Department of Integrated Traditional Chinese and Western Medicine, Rare Diseases Center, West China Hospital, Sichuan University, Chengdu, Sichuan 610041, China; State Key Laboratory of Biotherapy, Department of Integrated Traditional Chinese and Western Medicine, Rare Diseases Center, West China Hospital, Sichuan University, Chengdu, Sichuan 610041, China; State Key Laboratory of Biotherapy, Department of Integrated Traditional Chinese and Western Medicine, Rare Diseases Center, West China Hospital, Sichuan University, Chengdu, Sichuan 610041, China

**Keywords:** structural protein, MERS-CoV, SARS-CoV, SARS-CoV-2, X-ray crystallography, protein interaction

## Abstract

The membrane (M) protein is the most abundant structural protein of coronaviruses including MERS-CoV, SARS-CoV, and SARS-CoV-2, and plays a central role in virus assembly through its interaction with various partner proteins. However, mechanistic details about how M protein interacts with others remain elusive due to lack of high-resolution structures. Here, we present the first crystal structure of a betacoronavirus M protein from *Pipistrellus* bat coronavirus HKU5 (batCOV5-M), which is closely related to MERS-CoV, SARS-CoV, and SARS-CoV-2 M proteins. Furthermore, an interaction analysis indicates that the carboxy-terminus of the batCOV5 nucleocapsid (N) protein mediates its interaction with batCOV5-M. Combined with a computational docking analysis an M–N interaction model is proposed, providing insight into the mechanism of M protein–mediated protein interactions.

**Significance Statement**

M protein is the most abundant structural protein of coronaviruses including MERS-CoV, SARS-CoV, and SARS-CoV-2. It functions mainly through its interaction with other proteins, which is critical in virus assembly. In this study, the authors report the first atomic-resolution crystal structure of an M protein from a bat betacoronavirus and propose an interaction model between M and N proteins. These results provide structural insight into the mechanism of M protein-mediated protein interactions.

## Introduction

Coronaviruses are single-stranded RNA viruses that cause infections in animals and human (1, 2), and are divided into four genera: *Alphacoronavirus*, *Betacoronavirus*, *Gammacoronavirus*, and *Deltacoronavirus* (ICTV, http://www.ictvonline.org). To date, only seven coronaviruses are known to cause human illness (2). Two of them are alphacoronaviruses (HCoV-NL63 and HCoV-229E), and the rest belong to the *Betacoronavirus* genus, including MERS-CoV, SARS-CoV, and SARS-CoV-2 that have caused severe epidemic or pandemic in the last two decades (2, 3). Meanwhile, studies suggest that MERS-CoV, SARS-CoV, and SARS-CoV-2 all have bat origins (2, 4). For example, *Tylonycteris* bat coronavirus HKU4 and *Pipistrellus* bat coronavirus HKU5 are bat betacoronaviruses that are closely related to MERS-CoV in the *Merbecovirus* subgenus, and to a lesser extent, SARS-CoV and SARS-CoV-2 in the *Sarbecovirus* subgenus (5, 6).

The coronavirus virion contains four structural proteins: S (spike), M (membrane), E (envelope), and N (nucleocapsid) proteins (1, 7–9). Among them, M protein is the most abundant protein component and plays a central role in virus assembly mainly as a scaffolding center (1, 10–12). In coronaviruses, M protein is the only multispanning transmembrane structural protein and has been shown to interact with various partner proteins, including all viral structural proteins (S, M, E, and N) (1, 11, 13–20) and some host factors involved in the interferon signaling pathway such as MAVS (21–24). However, how M protein interacts with its partners remains largely unknown due to lack of high-resolution structures of M protein (25–28). In this study, we aim to determine the crystal structure of a coronavirus M protein, and to explore the interaction between M and N proteins in a structural context.

## Results and discussion

### Structure determination of a bat betacoronavirus M protein

To obtain a suitable M protein for structural study, we screened expression of M protein genes from eight betacoronavirus strains (1, 29), including MERS-CoV, SARS-CoV and SARS-CoV-2 (abbreviated as MERS, SARS and SARS2, respectively). Among them, the M protein from *Pipistrellus* bat coronavirus HKU5 (bat betacoronavirus M protein, batCOV5-M) displayed the best expression and biochemical properties (Fig. S1A), and was selected for further structural study. The batCOV5-M protein is closely related to MERS-M, and to a lesser extent, SARS-M and SARS2-M, sharing 95, 75 and 74% sequence homology, respectively (Fig. S2).

After extensive crystallization trials and refinement, batCOV5-M crystallized in lipidic cubic phase (LCP) and the crystals diffracted to ∼3 Å resolution. However, molecular replacement (30) using an AlphaFold (31)-predicted SARS2-M model (termed SARS2-M_AF_) (26) failed to yield a valid solution, suggesting that SARS2-M_AF_ may vary significantly from the experimental M structure. Therefore, we sought to resolve the phase problem by experimental means, such as single-wavelength anomalous diffraction using heavy atoms (32). Since batCOV5-M crystals grew only in the LCP glass sandwich (33), which makes soaking the crystals with heavy atoms impossible, we attempted to co-crystallize batCOV5-M with heavy atoms, but unfortunately unsuccessful. Our next strategy was to crystallize the cytosolic carboxy-terminal domain (CTD) of batCOV5-M (batCOV5-M_CTD_; Fig. S2) and solve its structure, which may serve as a searching template for molecular replacement of the full-length batCOV5-M.

**Fig. 1.**
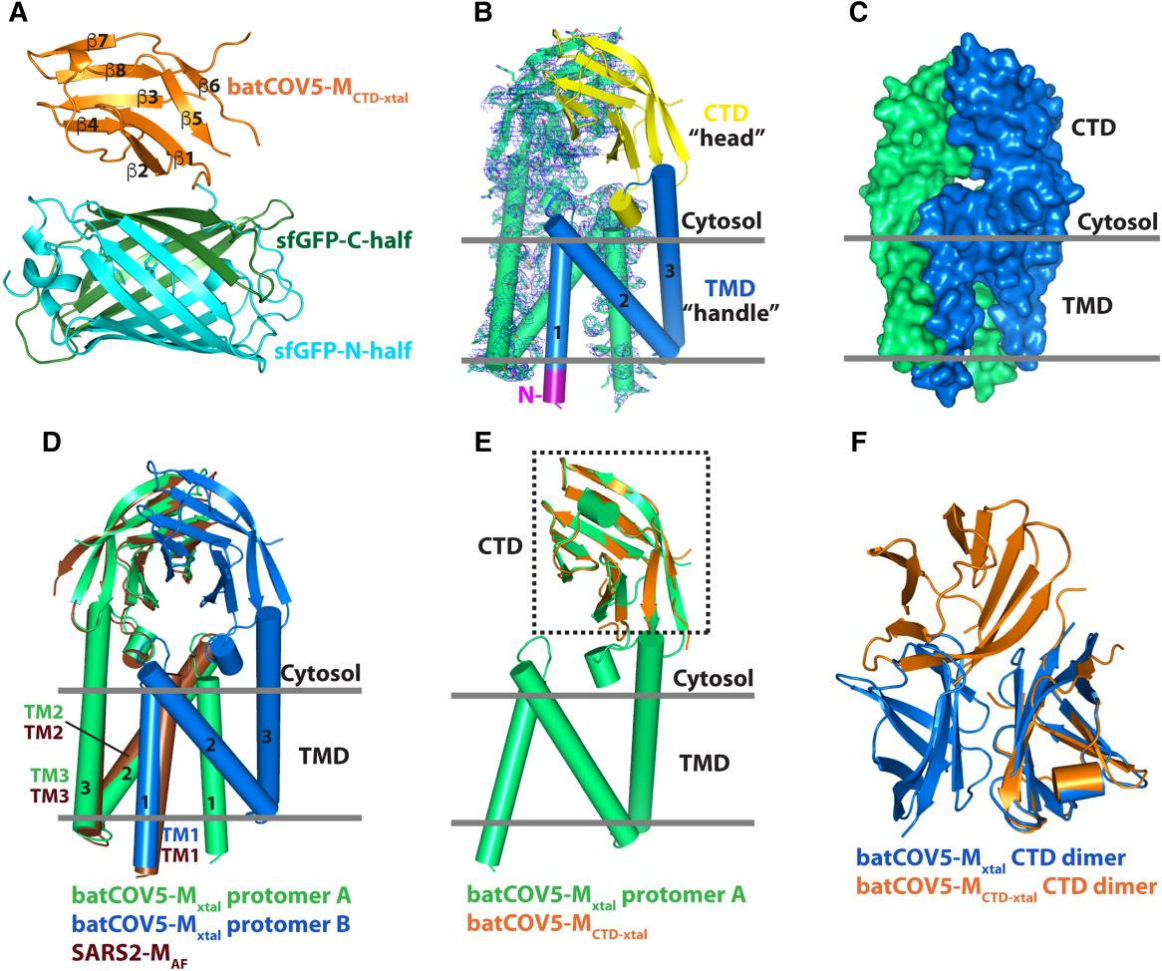
Crystal structure of M protein of the betacoronavirus batCOV5. A) Crystal structure of batCOV5-M_CTD_ (in orange) fused with a split superfolder GFP (N-terminal half in cyan and C-terminal half in dark green) rendered in the cartoon mode. The β strands of CTD are labeled from β1 to β8. B) Crystal structure of batCOV5-M homodimer. For one protomer (in green), the 2F_o_-F_c_ map contoured at 1.2 σ level is shown as blue mesh. In the other protomer, the N-terminus, TMD and CTD are displayed in purple, marine blue and yellow, respectively. The TM helices are labeled from 1 to 3 throughout this manuscript. The “head” and “handle” labels refer to an analogy to a pair of pincers in the main text. Relative membrane position is indicated by two gray lines throughout this manuscript. C) The batCOV5-M_xtal_ dimer is rendered as surfaces with one protomer in green and the other in marine blue. D) Superposition of the AlphaFold-predicted SARS2-M_AF_ monomer model (in brown) onto protomer A (in green) of batCOV5-M_xtal_ dimer. The batCOV5-M_xtal_ protomer B is displayed in marine blue. E) Superposition of batCOV5-M_CTD-xtal_ (in orange) onto one protomer of batCOV5-M_xtal_ (in green). F) Superposition of batCOV5-M_CTD-xtal_ dimer (in orange) onto CTD of the batCOV5-M_xtal_ dimer (in marine blue).

To express batCOV5-M_CTD_, we first fused the green fluorescent protein (GFP) moiety to the amino-terminus of the M_CTD_ domain (GFP-batCOV5-M_CTD_), similar to a previous SARS2-M study (34). However, the recombinant GFP-batCOV5-M_CTD_ appeared in the pellet fraction as indicated by GFP fluorescence and failed to be purified as a soluble protein. In the end, by fusing the two halves of a split superfolder GFP (sfGFP) (35, 36) to the two termini of batCOV5-M_CTD_, the resulting batCOV5-M_CTD_-sfGFP was crystallized by the vapor-diffusion sitting-drop method and its structure solved to 3.42 Å (Fig. S1B and Table 1) by molecular replacement using the GFP structure (PDB: 2B3Q) as a searching model. Interestingly, four batCOV5-M_CTD_-sfGFP molecules assemble into a tetramer in the structure (Fig. S1C), consistent with its oligomeric state in solution as verified by a cross-linking experiment (Fig. S1B). In the batCOV5-M_CTD_-sfGFP structure, the M_CTD_ domains swapping β strands with each other, resembling a “butterfly” shape (Fig. S1D). Three monomer conformations of M_CTD_ were then extracted from the tetramer: conformer 1 with the β sandwich split into three parts (Fig. S1E), conformer 2 with two parts (Fig. S1F), and conformer 3 as an assembled M_CTD_ (batCOV5-M_CTD-xtal_; Figs. 1A and S1G). Though it is tempting to postulate that domain swapping of CTD may play a role in regulating the M–M protein interaction (1), further investigation is required. Meanwhile, we tried to solve the full-length batCOV5-M structure by molecular replacement using the three CTD conformations as templates, and eventually succeeded with the batCOV5-M_CTD-xtal_ structure. The final batCOV5-M model was determined to 3.21 Å (batCOV5-M_xtal_; Fig. 1B and Table 1) and contained residues 13–199 of batCOV5-M, and the amino-terminus (12 residues) and the carboxy-terminus (21 residues) were not modeled due to weak electron densities.

**Table 1.** Data collection and refinement statistics

	batCOV5-M_CTD_-sfGFP	batCOV5-M_xtal_
PDB ID	7Y96	7Y9B
*Data collection*		
Space group	*P 6_1_ 2 2*	*P 1 2_1_ 1*
Wavelength (Å)	0.97915	0.97915
Unit cell *a, b, c* (Å) *α, β, γ* (°)	82.02, 82.02, 427.84 90, 90, 120	76.57, 66.57, 112.63 90, 109.83, 90
Resolution (Å)	3.42 (3.54–3.42)	3.21 (3.33–3.21)
Unique reflections	11,763 (1138)	16,670 (1745)
Multiplicity	4.4 (4.6)	3.4 (3.4)
Completeness (%)	93.77 (96.11)	93.93 (98.75)
*I/σI*	9.63 (3.09)	7.42 (1.76)
*R*_merge_	0.147 (0.508)	0.141 (0.694)
*R*_meas_	0.166 (0.569)	0.167 (0.827)
*R*_pim_	0.075 (0.252)	0.090 (0.444)
CC_1/2_	0.99 (0.841)	0.994 (0.74)
*Refinement*		
Resolution (Å)	3.42 (3.76–3.42)	3.21 (3.46–3.21)
No. reflections	11,762	16,586
Completeness (%)	93.9 (95.9)	94.0 (93.8)
*R*_work_ */R*_free_ (%)	24.0/26.5	27.1/29.3
No. atoms	4,890	6,039
Protein	4,846	5,939
Ligands	44	96
Solvent		4
Average *B-factor*	65	58
Protein	65	58
Ligands	53	52
Solvent		56
*Ramachandran*		
Favored (%)	97.54	98.38
Allowed (%)	2.46	1.62
Outliers (%)	0.00	0.00
RMS bonds (Å)	0.003	0.002
RMS angles (°)	0.658	0.412
Clashscore	8.73	6.70

Statistics for the highest resolution shell are shown in parentheses.

### The crystal structure of batCOV5-M_xtal_

The batCOV5-M_xtal_ structure is organized as a homodimer, with each protomer containing a short amino-terminus, a three-helix transmembrane domain (TMD) and a cytosolic CTD consisting of an eight-stranded β-sandwich (Fig. 1B). The overall shape of the batCOV5-M_xtal_ dimer looks like a pair of pincers, with TMD as the “handle” and CTD as the “head” of the pincers (Fig. 1B). The two protomers form extensive TMD–TMD and CTD–CTD contacts with a buried area of ∼3,100 Å^2^ at the dimer interface, which accounts for ∼25% of the solvent-accessible surface area of each protomer, suggesting that the batCOV5-M_xtal_ dimer is a stable form (Fig. 1C). In addition to the dimer interface, crystal packing analysis also reveals additional inter-dimer contacts between batCOV5-M_xtal_ dimers (Fig. S3A), including a “head-to-head” interaction via CTD (Fig. S3B), an “opposite” packing via TMD (Fig. S3C) and a “side-by-side” packing via CTD (Fig. S3D). These inter-dimer interactions suggest that higher order oligomers of M protein may occur in solution, as observed previously (37). Interestingly, cross-linking of batCOV5-M in solution using glutaraldehyde showed a small fraction of cross-linked batCOV5-M bands in protein gels corresponding to higher oligomeric species in addition to dimer (Fig. S1A). Nonetheless, the physiological relevance of these inter-dimer interactions to the function of M protein requires further investigation.

Not surprisingly, superposition of SARS2-M_AF_ onto one protomer of the batCOV5-M_xtal_ structure yielded an all-C_α_ RMSD of 4.4 Å with the major difference in TM1 (Fig. 1D). In the batCOV5-M_xtal_ structure, TM1 is placed almost in the sample plane as TM2/TM3, and the three helices of TMD resemble a capital letter “N,” while in SARS2-M_AF_ the TMD helices form a more compact three-helix bundle. As a result, the two TM1 helices swap positions in the batCOV5-M_xtal_ dimer compared with SARS2-M_AF_. Meanwhile, alignment between the batCOV5-M_CTD-xtal_ structure and CTD of the batCOV5-M_xtal_ structure yielded an all-atom RMSD of only 0.5 Å, indicating that the individually expressed CTD of batCOV5-M protein preserves its structure (Fig. 1E). This result may explain why molecular replacement for batCOV5-M_xtal_ failed when using SARS2-M_AF_ as a template, but succeeded with the batCOV5-M_CTD-xtal_ structure. However, it is noteworthy that the CTD dimer interface in the batCOV5-M_CTD_-sfGFP structure is different from that in the batCOV5-M_xtal_ structure (Fig. 1F).

### Comparison of the batCOV5-M and SARS2-M structures

**Fig. 2.**
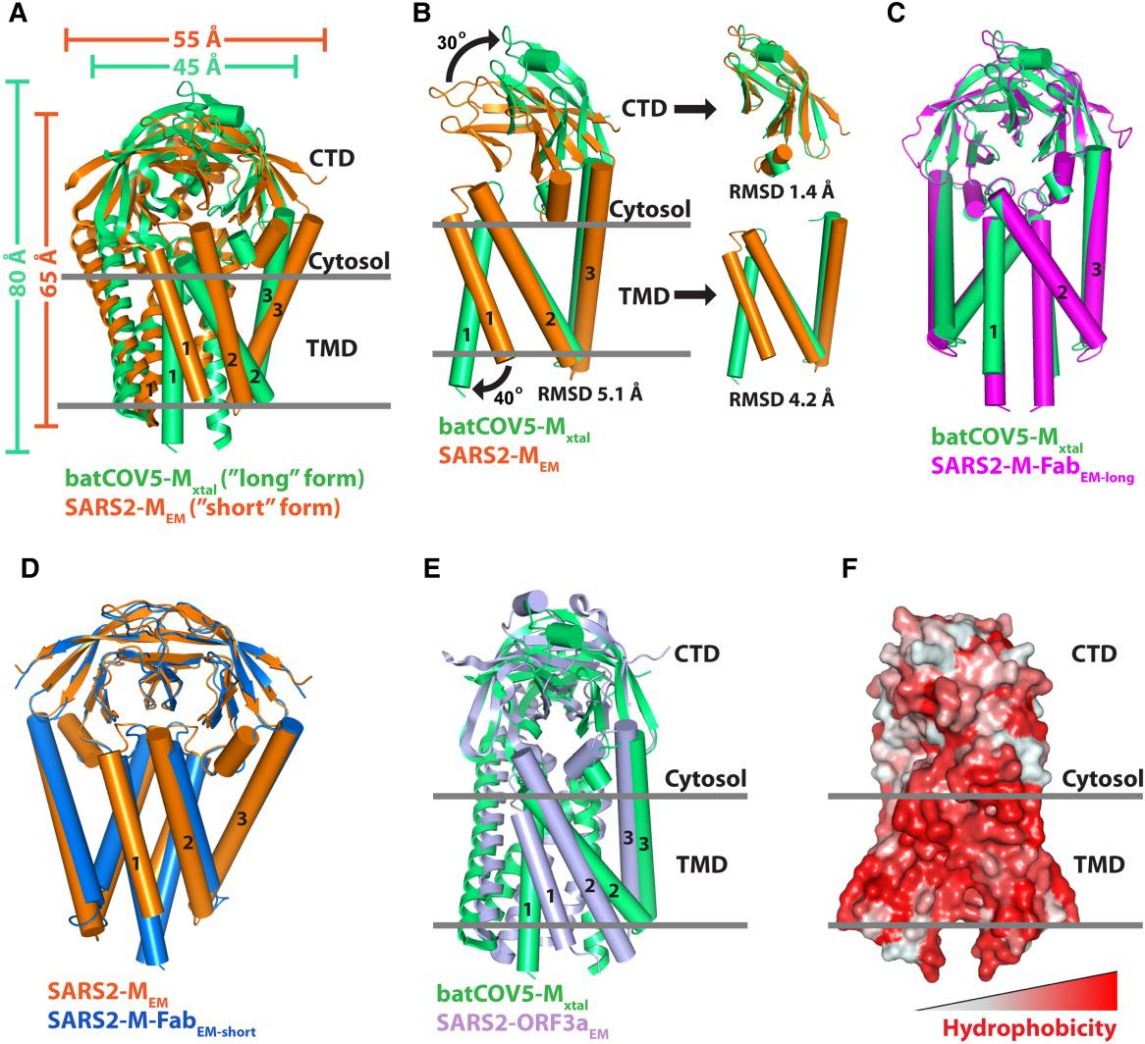
Comparison of the batCOV5-M_xtal_ structure and related structures. A) Superposition of the cryo-EM structure of SARS2-M_EM_ (PDB: 8CTK, in orange) onto batCOV5-M_xtal_ dimer (in green). For better viewing, each structure has one protomer rendered as cylinders. Dimensions of the two dimers are indicated. B) Left: Superposition of one SARS2-M_EM_ protomer (in orange) onto one batCOV5-M_xtal_ protomer (in green) using TM2/TM3 as anchors. Right: Individual CTDs and TMDs of the two structures are aligned using C_α_ atoms. C) Superposition of the SARS2-M-Fab_EM-long_ structure (PDB: 7VGR, in magenta) onto batCOV5-M_xtal_ dimer (in green). The Fab was not shown. D) Superposition of the SARS2-M-Fab_EM-short_ structure (PDB: 7VGS, in marine blue) onto SARS2-M_EM_ (PDB: 8CTK, in orange). The Fab was not shown. E) Superposition of the cryo-EM structure of SARS2-ORF3a_EM_ (PDB: 6XDC, in light blue) onto batCOV5-M_xtal_ dimer (in green). For better viewing, each structure has one protomer rendered as cylinders. F) Surface representation of batCOV5-M_xtal_ dimer based on the Eisenberg hydrophobicity scale.

During the submission of this manuscript, three cryo-EM structures of SARS2-M have also been reported by Dolan et al. (38, 39) and Zhang et al. (37). Dolan and colleagues first reported a cryo-EM structure of SARS2-M (SARS2-M_EM_, PDB: 8CTK) in bioRxiv (38). As expected, SARS2-M_EM_ is also a homodimer and shares a similar fold to the batCOV5-M_xtal_ structure (Fig. 2A). Intriguingly, superposition of SARS2-M_EM_ dimer onto batCOV5-M_xtal_ dimer yielded a large all-C_α_ RMSD of 6.3 Å, suggesting that the two structures are in different conformations. Alignment of the two dimer structures showed an apparent difference in shape, with batCOV5-M_xtal_ dimer long and slim (∼80 × 45 Å) while SARS2-M_EM_ dimer short and fat (∼65 × 55 Å) (Fig. 2A). This observation is consistent with the “long” and “short” conformations suggested previously (14), which may be involved in M–N interaction and virus assembly. To further analyze the conformational change between SARS2-M_EM_ and batCOV5-M_xtal_, we superimposed single protomers of the two structures, yielding an all-C_α_ RMSD of 5.1 Å, whereas alignment of single CTDs and single TMDs between the two structures yielded all-C_α_ RMSDs of 1.4 and 4.2 Å, respectively (Fig. 2B). Comparison of single protomers between SARS2-M_EM_ and batCOV5-M_xtal_ reveals that their CTDs are highly alike, but their TMDs differ significantly in TM1, which is much closer to TM2 in SARS2-M_EM_ than in batCOV5-M_xtal_ (Fig. 2B). As a result, using TM2/TM3 as anchors, TM1 swings ∼40 degrees away from TM2 and CTD undergoes a rigid-body rotation of ∼30 degrees away from TMD during a transition from the “short” SARS2-M_EM_ to the “long” batCOV5-M_xtal_ (Fig. 2B). Meanwhile, the two protomers move ∼10 Å closer (Fig. 2A).

Interestingly, in the meantime, Zhang and colleagues reported two cryo-EM structures of SARS2-M in complex with specific Fab fragments (37), representing a “long” form (SARS2-M-Fab_EM-long_, PDB: 7VGR) and a “short” form (SARS2-M-Fab_EM-short_, PDB: 7VGS). Structural alignment yielded an all-C_α_ RMSD of 1.8 Å between batCOV5-M_xtal_ and SARS2-M-Fab_EM-long_ (Fig. 2C) and an all-C_α_ RMSD of 1.6 Å between SARS2-M_EM_ and SARS2-M-Fab_EM-short_ (Fig. 2D), while the major deviation came from flexible loops of these proteins (Fig. 2C and D). This result indicates that the structural organization is highly conserved in betacoronavirus M proteins, and the batCOV5-M_xtal_ structure represents the long conformation of M protein.

Intriguingly, a structural comparison analysis using the Dali server (40) indicates the batCOV5-M_xtal_ structure also shares fold with the cryo-EM structure of SARS-CoV-2 ORF3a protein (SARS2-ORF3a_EM_, PDB: 6XDC), which has previously been observed for SARS2-M structures as well (37, 39). The SARS2-ORF3a_EM_ structure is also a homodimer (41). Indeed, superposition of the two dimer structures yielded an all-C_α_ RMSD of 3.8 Å, suggesting that the overall folding of the two structures is alike (Fig. 2E). Meanwhile, SARS2-ORF3a has been suggested to be a cation channel with a polar permeation pathway (41). However, there is few polar or charged residues present in the batCOV5-M TMD (Figs. 2F and S2), making it unlikely also a cation channel as SARS2-ORF3a. Similarly, studies have also suggested that SARS2-M does not function as an ion channel (37, 39). It is worth noting that a recent study called into question the validity of SARS2-ORF3a being an ion channel (42), suggesting that more investigation may be required to address this issue.

### Exploring the M–N interaction of batCOV5 by a pull-down assay

**Fig. 3.**
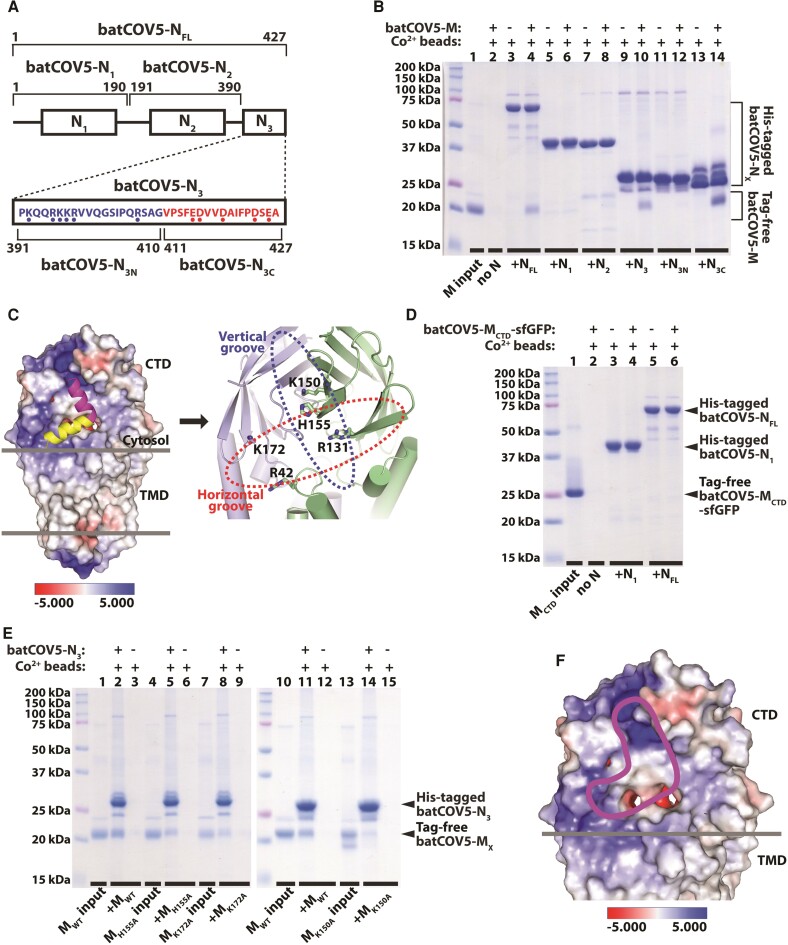
Probing the M–N interaction of batCOV5 in a structural context. A) Scheme of batCOV5-N fragments used in this study. The N_3N_ and N_3C_ sequences are shown with blue or red dots indicating basic or acidic residues, respectively. B) A pull-down experiment using purified His-tagged batCOV5-N fragments to pull-down tag-free batCOV5-M protein is examined by SDS-PAGE analysis. Signs and labels above and below the gel indicate the components present in the pull-down experiment. The label “batCOV5-N_X_” refers to all batCOV5-N fragments in this panel. Lane 1 is the batCOV5-M protein input for pull-down. Molecular weight of protein standards is labeled on the left of gel. C) Left: Two docked models of batCOV5-N_3C_ (as a yellow helix and a magenta helix, respectively) into the batCOV5-M_xtal_ dimer structure, which is displayed as an electrostatics surface. Right: An enlarged view of the “horizontal groove” (dashed red oval) and the “vertical groove” (dashed blue oval). Five residues within the two grooves are rendered as sticks. D) A pull-down experiment using purified His-tagged batCOV5-N fragments to pull-down tag-free batCOV5-M_CTD_-sfGFP protein, similar to panel (B). E) A pull-down experiment using purified His-tagged batCOV5-N_3_ to pull-down tag-free wild-type or mutant batCOV5-M proteins, similar to panel (B). The label “batCOV5-M_X_” refers to all batCOV5-M variants in this panel. F) A potential binding groove for batCOV5-N_3C_ is indicated by a magenta outline on the electrostatics surface of batCOV5-M_xtal_. For the scale of electrostatics surface, red indicates negatively charged surface, while blue indicates positively charged surface. All pull-down experiments were repeated three times using biologically independent samples with similar results.

To validate the batCOV5-M_xtal_ structure and to study its function, we then investigated the interaction between M and N proteins of batCOV5-M. The coronavirus M–N interaction has been shown to facilitate virion assembly and budding of virus-like particles (11, 12, 18–20). N protein is an RNA-binding protein containing an amino-terminal RNA-binding domain (N_1_) and a carboxy-terminal dimerization domain (N_2_) (18–20, 34) (Fig. 3A). An N_3_ region at the end of the carboxy terminus has been shown to interact with M protein (18–20). Sequence alignment indicates that N_1_ and N_2_ are highly conserved among betacoronaviruses, whereas N_3_ is more variable (Fig. S4). Therefore, we generated four constructs for expressing N protein and its fragments: the full-length protein (residues 1–427, batCOV5-N_FL_), the N_1_ fragment (residues 1–190, batCOV5-N_1_), the N_2_ fragment (residues 191–390, batCOV5-N_2_), and the N_3_ fragment (residues 391–427, batCOV5-N_3_) (Fig. 3A). In a pull-down analysis, purified batCOV5-N_FL_ and batCOV5-N_3_ proteins, but not batCOV5-N_1_ or batCOV5-N_2_, displayed robust interactions with purified wild-type batCOV5-M protein (Fig. 3B, lanes 4 and 10), consistent with previous reports (18–20).

It has also been suggested that the N_3_ region may interact with M protein through electrostatic interactions (37, 43–45). Interestingly, batCOV5-N_3_ contains six basic residues in its amino-terminal half (residues 391–410, batCOV5-N_3N_) and five acidic residues in its carboxy-terminal half (residues 411–427, batCOV5-N_3C_) (Fig. 3A). Although the N_3_ sequence is not very conserved among betacoronaviruses, the pattern of “basic-amino-half-and-acidic-carboxy-half” for the N_3_ region seems to be a conserved feature in *Betacoronavirus* including MERS-CoV, SARS-CoV and SARS-CoV-2 (Fig. S4). The pull-down analysis clearly showed that purified batCOV5-N_3C_, but not batCOV5-N_3N_, pulled M protein down (Fig. 3B, lane 14). Furthermore, single mutations at the five acidic residue positions (E415, D416, D419, D424, and E426) of batCOV5-N_3_ decreased its binding affinity for batCOV5-M to varying extent in a microscale thermophoresis assay (MST; Table S1). This result supports the notion that the negatively charged batCOV5-N_3C_ interacts with the Lys/Arg/His-rich CTD of batCOV5-M (Figs. 3C and S2), and charge interactions may play an important role in this process. Consistently, this notion is also supported by a recent study of the M–N (and RNA) interaction of SARS-CoV-2 (37).

### Probing the M–N interaction of batCOV5 in a structural context

**Fig. 4.**
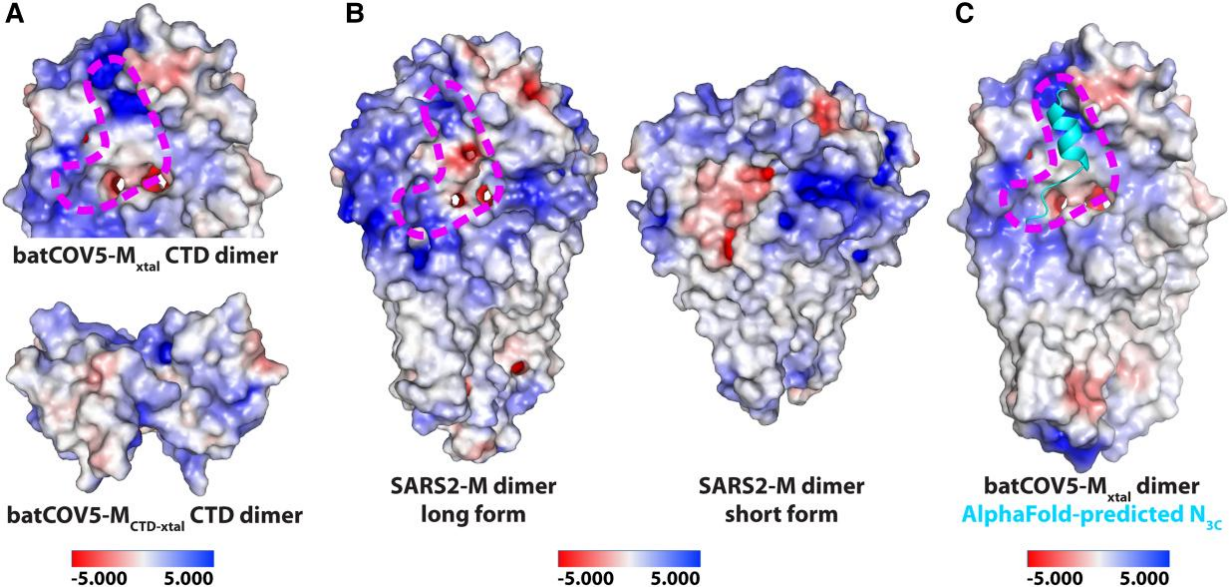
Formation of the potential binding groove for N_3C_ in M protein. A) Electrostatics surface of the CTD dimer in the batCOV5-M_xtal_ structure (top) and in the batCOV5-M_CTD_-sfGFP structure (bottom). A dashed magenta outline indicates a potential binding groove for N_3C_ throughout this figure. B) Electrostatics surface of the long form (left, PDB: 7VGR) and the short form (right, PDB: 8CTK) of SARS2-M dimer structures. C) A more flexible model of batCOV5-N_3C_ (in cyan) predicted by AlphaFold is manually fitted in the potential binding groove on the batCOV5-M_xtal_ structure. For the scale of electrostatics surface, red indicates negatively charged surface, while blue indicates positively charged surface.

To further explore the binding mechanism of M protein and the N_3C_ fragment, we created docking models (see Methods for details) using the batCOV5-M_xtal_ dimer structure and the batCOV5-N_3C_ sequence through the HDOCK web server (46, 47). HDOCK predicted batCOV5-N_3C_ as a helix and generated two plausible binding patterns between batCOV5-M_xtal_ and batCOV5-N_3C_ that are consistent with the electrostatic potential surface of the batCOV5-M_xtal_ dimer (Fig. 3C). In the first pattern, the N_3C_ helix binds at the interface between the CTD dimer and the TMD dimer, which form a groove nearly horizontal to the membrane plane. In the second pattern, the N_3C_ helix binds to a groove formed along the CTD dimer interface nearly vertical to the membrane plane. Both binding patterns require dimerization of batCOV5-M to form a positively charged groove to accommodate the negatively charged N_3C_ (Fig. 3C). This result may explain why purified batCOV5-M_CTD_-sfGFP did not bind batCOV5-N_FL_ (Fig. 3D), because the M_CTD_ dimer in the batCOV5-M_CTD_-sfGFP structure does not form a positively charged groove as in the batCOV5-M_xtal_ structure (Fig. 4A). Interestingly, a structural analysis of SARS2-M revealed that the short conformation of SARS2-M also forms a very different dimer interface than the long form, and destroys the positively charged groove formed in the long conformation (Fig. 4B). This result suggests that the conformation of M protein may affect its ability to bind N protein, which has been observed previously in mouse hepatitis virus (also a betacoronavirus) that only the long form of M protein is associated with N protein (14).

Nonetheless, which binding pattern between batCOV5-M_xtal_ and batCOV5-N_3C_ may be correct? To gain more insights, the basic residues within the two putative binding grooves of batCOV5-M were selected for mutagenesis. For the horizontal groove Arg42 and Lys172 were selected, whereas Lys150 was selected specifically for the vertical groove (Fig. 3C). Meanwhile, Arg131 and His155 were selected for both grooves as the two residues are located at the crossing of the two grooves (Fig. 3C, right panel). Unfortunately, although Arg42 and Arg131 are highly conserved among various betacoronaviruses (Fig. S2), both batCOV5-M mutants of R42A and R131A failed to express and could not be assessed. Interestingly, mutants of the other three less conserved residues (K150A, H155A, and K172A; Fig. S2) all displayed apparently less binding to batCOV5-N_3_ in pull-down assays (Fig. 3E), suggesting that binding of batCOV5-N_3C_ to batCOV5-M may involve residues from both grooves (Fig. 3F).

Therefore, several points are worth discussing. First, involvement of both groove residues in M–N binding suggests that the HDOCK models may not be sufficiently accurate, especially when a pure in silico structure (N_3C_ here) is involved. Indeed, AlphaFold-predicted batCOV5-N_3C_ as a different model with a flexible carboxy-terminus following a short helix, which may be capable of binding both grooves (Fig. 4C). Therefore, in silico structures or complex models need to be interpreted with caution and be validated with experimental data. Second, His155 is not charged under the pull-down assay condition (pH 7.5), suggesting that other interactions (e.g. hydrogen bonds) likely also contribute to M–N binding. To verify the H155A result, binding of batCOV5-M_H155A_ to batCOV5-N_3_ was measured by MST, which showed a decreased binding affinity (equilibrium dissociation constant *K*_d_ = 3.60 ± 0.17 μM) compared with the wild-type batCOV5-M protein (*K*_d_ = 0.77 ± 0.13 μM) (Table S1), consistent with the pull-down result. Third, participation of the less conserved residues (Lys150, His155, and Lys172) of batCOV5-M in M–N binding suggests that the binding patterns between M and N proteins may not be very conserved in various betacoronaviruses. This notion is also consistent with the fact that the N_3C_ region is not very conserved in *Betacoronavirus* (Fig. S4), suggesting that each betacoronavirus may employ a slightly different set of key residues to mediate the M–N interaction. Further investigation, especially the M–N complex structures from different betacoronaviruses, will help address this issue in the future.

## Methods

### Protein expression and purification

The gene encoding M protein of *Pipistrellus* bat coronavirus HKU5 (batCOV5-M, NCBI accession YP_001039968.1) was synthesized (Genewiz, China) and cloned into a modified pPICZ plasmid (Thermo Fisher Scientific) containing an N-terminal tag of FLAG-His_10_-TEV protease recognition site. All batCOV5-M mutations were introduced by QuikChange II system (Agilent) according to manufacturer's recommendation, and all mutations were verified by DNA sequencing. The constructs were linearized and transformed into *Pichia pastoris* strain GS115 by lithium chloride/single-strand carrier DNA/polyethylene glycol method according to manufacturer's manual (Thermo Fisher Scientific). The transformants were inoculated into YPD medium consisting of 1% (w/v) yeast extract, 2% (w/v) peptone and 2% (w/v) D-(+)-glucose at 30°C with shaking at 220 rpm until an OD_600_ of 3–5 was reached. To induce protein expression, yeast cells were harvested by centrifugation and resuspended to an OD_600_ of 1 in YPM medium consisting of 1% (w/v) yeast extract, 2% (w/v) peptone, 0.8% (v/v) methanol, and 2.5% (v/v) dimethyl sulfoxide at 30°C for 24 h. Cell pellets were resuspended in Lysis Solution (LS) containing 20 mM Tris-HCl pH 7.5, 150 mM NaCl, 10% (v/v) glycerol, 1 mM phenylmethanesulfonyl fluoride (PMSF) and 2 mM β-mercaptoethanol, and were lysed by an AH-1500 high-pressure homogenizer (ATS, China) at 1,300 MPa. Undisrupted cells and cell debris were separated by centrifugation at 3,000*×g*, and membrane was collected by ultracentrifugation at 140,000*×g* for 1 h at 4°C. Protein was extracted by addition of 1% (w/v) *n*-dodecyl-β-D-maltopyranoside (DDM; Anatrace) at 4°C for 2 h and the extraction mixture was centrifuged at 200,000*×g* for 30 min at 4°C. The supernatant was incubated with Co^2+^ resin in the presence of 20 mM imidazole pH 8.0 at 4°C for 1 h, and the mixture was loaded in an empty chromatography column. The resin/protein was washed with 20 bed-volume of LS containing 2 mM DDM and 30 mM imidazole pH 8.0, and the protein was eluted with LS supplemented with 2 mM DDM and 250 mM imidazole pH 8.0.

To generate the GFP-batCOV5-M_CTD_ construct, the DNA sequence encoding batCOV5-M_CTD_ (residues 115–203 of batCOV5-M) was amplified by polymerase chain reaction (PCR) and cloned into a modified pPICZ plasmid (Thermo Fisher Scientific) containing a N-terminal His_10_-TEV site-GFP tag. To generate the batCOV5-M_CTD_-sfGFP construct, a superfolder GFP (35) was split into two halves (21) and was fused to the N- and C-termini of batCOV5-M_CTD_ (residues 115–203 of batCOV5-M) by gene synthesis (Genewiz, China). The fusion protein-encoding DNA was cloned into a modified pPICZ plasmid (Thermo Fisher Scientific) containing a C-terminal TEV site and a His_10_ tag. Transformation and expression of GFP-batCOV5-M_CTD_ and batCOV5-M_CTD_-sfGFP followed the same protocol as batCOV5-M except that the expression was induced at 25°C. Cell pellets were resuspended in LS containing 20 mM Tris-HCl pH 8.0, 150 mM NaCl, 20% (v/v) glycerol and 1 mM PMSF, and were lysed similarly to batCOV5-M. Undisrupted cells and cell debris were separated by centrifugation at 140,000*×g* at 4°C for 1 h. The supernatant was supplemented with 20 mM imidazole pH 8.0 and was immediately loaded onto a pre-washed Co^2+^ affinity column. The column was then washed with 20 bed-volume of LS containing 30 mM imidazole pH 8.0, and the protein was eluted with LS containing 250 mM imidazole pH 8.0.

The gene encoding N protein of batCOV5 (batCOV5-N, NCBI accession YP_001039969.1) was synthesized (Genewiz, China) and cloned into a modified pPICZ plasmid (Thermo Fisher Scientific) containing an N-terminal tag of FLAG-His_10_-TEV site, followed by the bacterial cytochrome b562RIL (BRIL) (48) to improve expression of the protein of interest. All batCOV5-N fragments were generated by PCR and cloned into the same vector as batCOV5-N. For MST analysis, batCOV5-N_3_-enconding DNA was subcloned into another modified pPICZ plasmid (Thermo Fisher Scientific) containing a C-terminal tag of TEV site-GFP-His_10_. Single mutations of batCOV5-N_3_ were introduced by QuikChange II system (Agilent). Transformation, expression, and purification of batCOV5-N and fragments followed the same protocol as batCOV5-M_CTD_-sfGFP, except that LS contained 20 mM Tris-HCl pH 7.5, 150 mM NaCl, 10% (v/v) glycerol and 1 mM PMSF.

### Crystallization

Affinity-purified batCOV5-M protein was concentrated to 5 mg/mL, and treated with trypsin (TPCK treated; Sigma-Aldrich) at a 1:50 ratio (trypsin:batCOV5-M, w/w) for 20 min at 18°C to generate a stable core. The digestion was stopped by 10 mM PMSF and the protein was further purified by size-exclusion chromatography (SEC) in a buffer consisting of 150 mM NaCl, 20 mM Tris-HCl pH 7.5, 0.5 mM DDM and 5 mM β-mercaptoethanol. The peak fractions were pooled, concentrated to ∼30 mg/mL, and mixed with 1-oleoyl-rac-glycerol (monoolein; Sigma-Aldrich) at a 2:3 ratio (protein:lipid, w/w) using the twin-syringe mixing method (33). The protein–lipid mixture was dispensed manually in ∼50 nL drops onto 96-well glass sandwich plates and overlaid with 0.8 μL of precipitant solution per drop. The batCOV5-M crystals were grown in 300 mM ammonium formate, 50 mM Tris-HCl pH 8.8, 35% (v/v) PEG 500 monomethyl ether and 7.1 mM pentaethylene glycol monooctyl ether. The crystals usually appear in 1 week and grow to full size in 2 weeks, and were flash-frozen directly in liquid nitrogen without additional cryoprotection.

For batCOV5-M_CTD_-sfGFP, affinity-purified protein was used directly for crystallization by vapor-diffusion sitting-drop method without further SEC purification or concentration step. The batCOV5-M_CTD_-sfGFP crystals were grown in 0.4 M ammonium sulfate, 0.1 M Bis-Tris pH 5.3, 27% (w/v) PEG 3350, and 0.5% (v/v) ethyl acetate. The crystals were either cryoprotected by 15–20% (v/v) glycerol or directly flash frozen in liquid nitrogen without additional cryoprotection.

### Data collection and structure solution

Diffraction data were collected on beamlines BL18U1 and BL19U1 (49) of National Facility for Protein Science in Shanghai (NFPS) at Shanghai Synchrotron Radiation Facility (SSRF). The data were indexed, integrated, and scaled using the autoPROC pipeline package (Global Phasing Limited) (50), which includes XDS (51) and AIMLESS (CCP4 package) (52). The batCOV5-M_CTD_-sfGFP structure was solved by molecular replacement with Phaser (53) using a published superfolder GFP structure (35) (PDB: 2B3Q) as a template. The full-length batCOV5-M structure was then solved by molecular replacement using the assembled batCOV5-M_CTD_ structure (Fig. S1G) as a searching model. Manual model building and refinement was carried out using Coot (54) and phenix.refine (55), and Molprobity (56) was used to monitor and improve protein geometry. Non-crystallographic symmetry restraints were applied throughout the refinement to improve maps. The data collection and refinement statistics were generated using phenix.table_one (55) and the values are listed in Table 1. All structural figures, RMSD calculations, and length/angle/area measurements were performed in PyMOL (Schrödinger, LLC). Hydrophobicity analysis was performed in PyMOL (Schrödinger, LLC) using a “color_h” script from PyMOLwiki (https://pymolwiki.org/index.php/Color_h) based on the Eisenberg hydrophobicity scale (57). The Adaptive Poisson-Boltzmann Solver (APBS) electrostatics calculation was performed using APBS Electrostatics Plugin within PyMOL (Schrödinger, LLC).

### Chemical cross-linking

Affinity-purified batCOV5-M and batCOV5-M_CTD_-sfGFP were further purified by SEC in a buffer consisting of 20 mM HEPES-Na^+^ pH 7.5, 150 mM NaCl and 0.5 mM DDM. Glutaraldehyde was added to protein samples to final concentrations of 0.5, 1, and 2 mM. The cross-linking reaction was carried out for 30 min at room temperature and was stopped by adding Tris-HCl pH 7.5 to a final concentration of 10 mM. The batCOV5-M_CTD_-sfGFP samples were heated to 95°C for 5 min to ensure a complete denaturation of the GFP moiety before the cross-linked samples being analyzed by SDS-PAGE gels.

### Pull-down assay

For pull-down assays, affinity-purified batCOV5-M and mutants were treated with TEV protease (to remove His-tag) and Endoglycosidase H (New England Biolabs), and purified batCOV5-M_CTD_-sfGFP was treated with TEV protease, then all were further purified by SEC. In a pull-down experiment, affinity-purified batCOV5-N and fragments were first incubated with Co^2+^ beads for 30 min at 4°C, followed by addition of His-tag-free batCOV5-M or variants to continue incubation for another 30 min at 4°C in the presence of 30 mM imidazole pH 8.0. The Co^2+^ beads were collected by centrifugation at 5,000*×g* for 1 min and washed twice with the assay buffer before being analyzed by SDS-PAGE gels.

### Docking of batCOV5-N_3C_ into the batCOV5-M_xtal_ structure

Computational docking was performed using the HDOCK web server (46) (http://hdock.phys.hust.edu.cn/). For the input of receptor, the batCOV5-M_xtal_ structure was prepared as a .pdb file containing only one protein dimer (chains A and B) by removing other chains and all “HETATM” records. For the input of ligand, non-polar residues at both ends of the batCOV5-N_3C_ sequence (VPSFEDVVDAIFPDSEA, 17 residues) were removed to generate a core sequence (SFEDVVDAIFPDSE, 14 residues), which was inputted directly in the HDOCK's ligand section in FASTA format. Alternatively, batCOV5-N_3C_ could also be prepared as a.pdb file using a predicted batCOV5-N structure by AlphaFold (https://alphafold.com) (31). Docking was performed in the template-free docking mode. Docked models were manually examined to remove incorrectly placed batCOV5-N_3C_ (e.g. being placed inside the membrane).

### Microscale thermophoresis

MST analysis was performed using Monolith NT.115 (NanoTemper, Germany). All affinity-purified proteins were further purified by SEC in a buffer containing 20 mM Tris-HCl pH 7.5, 150 mM NaCl and 0.5 mM DDM. The peak fractions were pooled and diluted to 100 nM using the SEC buffer. The GFP moiety fused to batCOV5-N_3_ and its mutants provided the fluorescence signal required by MST. Meanwhile, purified batCOV5-M and batCOV5-M_H155A_ served as ligands and were prepared according to the MST manual with the highest concentration of 32 μM for both proteins. The batCOV5-N_3_ and mutant samples were mixed with serial-diluted ligands and were incubated for 10 min at room temperature. Then the samples were loaded into capillaries and MST measurements were performed according to the Monolith manual. The equilibrium dissociation constant (*K*_d_) was determined in the MO.Affinity Analysis software (NanoTemper) with the *K*_d_ fit function. All MST measurements were performed in three biologically independent experiments (*N* = 3), and *K*_d_ values are expressed as mean ± SD in the text and Table S1. Two-tailed Student's *t*-test was performed for statistical analysis in Table S1.

## Supplementary Material

pgad021_Supplementary_Data

## Data Availability

The atomic coordinates and structure factors generated in this study have been deposited in the Protein Data Bank under the following accession codes: 7Y96 for batCOV5-M_CTD_-sfGFP and 7Y9B for batCOV5-M. Five previously reported structures used in this study are available in the Protein Data Bank under the following accession codes: 2B3Q for a superfolder GFP, 6XDC for ORF3a of SARS-CoV-2, and 7VGS, 7VGR, and 8CTK for M protein of SARS-CoV-2. Source data of *K*_d_ values are provided in Table S1.
