# Supplementary material for: Crystal structure of the membrane (M) protein from a bat betacoronavirus
Source: PNAS Nexus. 2023 Jan 30;2(2):pgad021. doi: 10.1093/pnasnexus/pgad021 (PMC9982069; doi:10.1093/pnasnexus/pgad021)
Supplement: pgad021_Supplementary_Data [file pgad021_supplementary_data.pdf]

Supplementary Material for

Crystal structure of the membrane (M) protein from a bat betacoronavirus

Xiaodong Wang[1], Yuwei Yang[1], Ziyi Sun[1]*, Xiaoming Zhou[1]*

[1]State Key Laboratory of Biotherapy, Department of Integrated Traditional Chinese and Western Medicine, Rare Diseases Center, West China Hospital, Sichuan University, Chengdu, Sichuan 610041, China

*To whom correspondence should be addressed:

Ziyi Sun, PhD, 17 Renmin Road South 3rd Section, Sichuan University, Chengdu, Sichuan 610041, China; Phone: +86 (28) 8540 1152; Email: ziyi.sun@scu.edu.cn

Xiaoming Zhou, PhD, 17 Renmin Road South 3rd Section, Sichuan University, Chengdu, Sichuan 610041, China; Phone: +86 (28) 8540 1152; Email: x.zhou@scu.edu.cn

This PDF file includes:

Table S1

Figures S1 to S4

Supplementary tables

Table S1. Affinities of batCOV5-M to batCOV5-N_3 determined by MST.

(K_d: μM)	Repeat 1	Repeat 2	Repeat 3	Mean	SD	*P*
**M WT/N_3 WT**	0.65	0.77	0.90	0.77	0.13	-
**M WT/N_3 E415A**	3.40	3.40	3.70	3.50	0.17	< 0.001
**M WT/N_3 D416A**	3.00	3.00	3.20	3.07	0.12	< 0.001
**M WT/N_3 D416N**	2.10	2.20	2.70	2.33	0.32	0.001
**M WT/N_3 D419A**	4.50	5.00	5.00	4.83	0.29	< 0.001
**M WT/N_3 D424A**	1.20	1.90	2.60	1.90	0.70	0.052
**M WT/N_3 E426Q**	2.80	3.40	3.80	3.33	0.50	0.001
**M H155A/N_3 WT**	3.40	3.70	3.70	3.60	0.17	< 0.001

All MST measurements were repeated with three biologically independent samples (N=3) and source data of K_d values are shown. Two-tailed Student's t-test was performed between the "M WT/N_3 WT" group and other groups, and P values are shown.

Supplementary figures

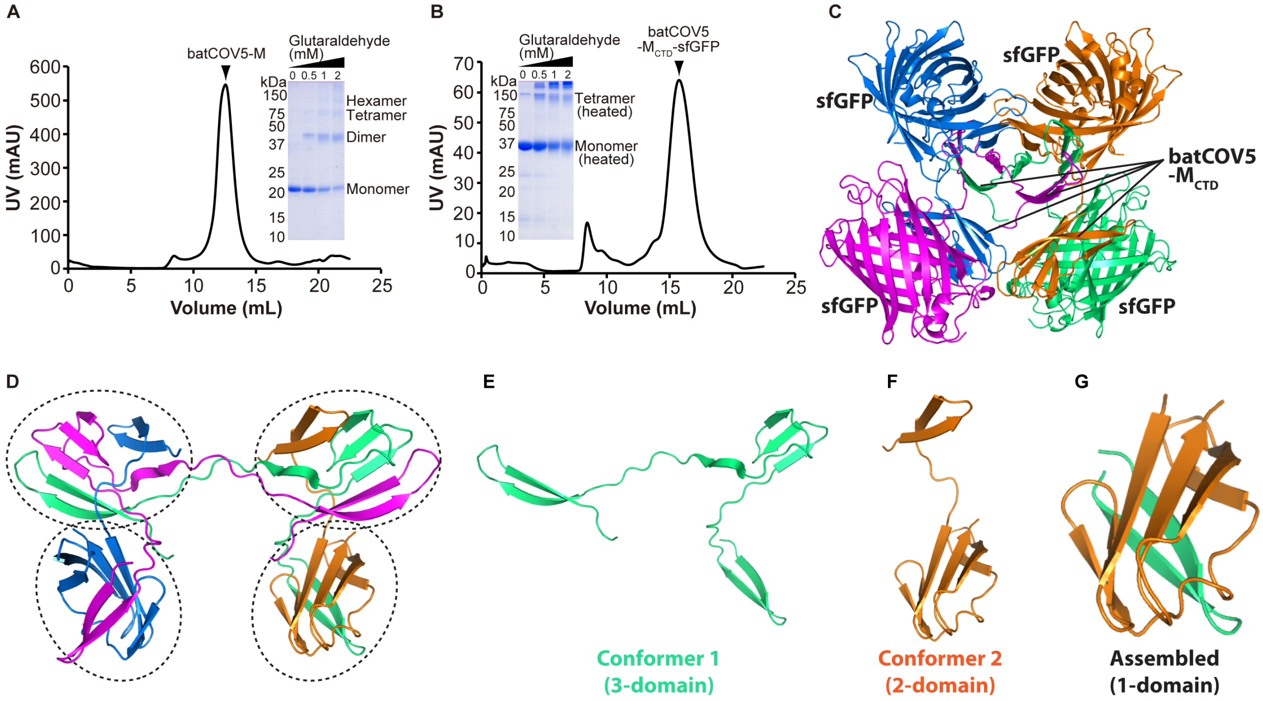

Figure S1. Preparative work for determining the structure of batCOV5-M. (A) Elution profile of purified batCOV5-M from a size exclusion chromatography. The inset shows the SDS-PAGE analysis of the batCOV5-M sample after being cross-linked by glutaraldehyde as indicated. (B) Elution profile of purified batCOV5-M_{CTD}-sfGFP from a size exclusion chromatography. The inset shows the SDS-PAGE analysis of the batCOV5-M_{CTD}-sfGFP sample after being cross-linked by glutaraldehyde and being heated as described in Methods. (C) Tetrameric batCOV5-M_{CTD}-sfGFP displayed in four colors. (D) The batCOV5-M_{CTD} tetramer in the batCOV5-M_{CTD}-sfGFP structure is formed by swapping β strands with each other. Each color indicates one protomer. Each dashed oval indicates one assembled batCOV5-M_{CTD}. The sfGFP structure is not displayed for clearer viewing. (E)-(G) Three conformations of batCOV5-M_{CTD}.

```
                         TM1                          
MERS-M      M-SNMTQLTEAQIIAIIKDWNFAWSLIFLLITIVLQYGYPSRSMTVYVFKMFVLWLLWPS   59
batCOV5-M   MASSNVTLSNDEVLRLVKDWNFTWSVVFLLITIVLQYGYPSRSMFVYVIKMFVLWLLWPA   60
SARS2-M     MADSNGTITVEELKKLLEQWNLVIGFLFLTWICLLQFAYANRNRFLYIIKLIFLWLLWPV   60
SARS-M      MAD-NGTITVEKLKQLLEQWNLVIGFLFLAWIMLLQFAYSNRNRFLYIIKLVFLWLLWPV   59
            * .    ::  ::  ::::**:. ..:**    :**:.*..*.  :*::*:..******

              TM2          TM3
MERS-M      SMALSIFSAIYPIDLASQIISGIVAAVSAMMWISYFVQSIRLFMRTGSWWSFNPETNCLL  119
batCOV5-M   SMALSIFCAVYPIDLASQIISGILAATSCAMWISYFVQSIRLFMRTGSWWSFNPESNCLL  120
SARS2-M     TLACFVLAAVYRINWITGGIAIAMACLVGLMWLSYFIASFRLFARTRSMWSFNPETNILL  120
SARS-M      TLACFVLAAVYRINWVTGGIAIAMACIVGLMWLSYFVASFRLFARTRSMWSFNPETNILL  119
            ::*  ::.*:* *:  :  *:  :*.    **:***: *:*** ** * ******:* **

MERS-M      NVPIGGTTVVRPLVEDSTSVTAVVTNGHLKMAGMHFGACDYDRLPNEVTVAKPNVLIALK  179
batCOV5-M   NVPIGGTTVVRPLVEDSTSVTAVVTDGYLKMAGMHFGACDFQRLPSEVTVAKPNVLIALK  180
SARS2-M     NVPLHGTILTRPLLESELVIGAVILRGHLRIAGHHLGRCDIKDLPKEITVATSRTLSYYK  180
SARS-M      NVPLRGTIVTRPLMESELVIGAVIIRGHLRMAGHSLGRCDIKDLPKEITVATSRTLSYYK  179
            ***: ** :.***:*..  : **:  *:*::**  :* ** . **.*:***....*   *

MERS-M      MVKRQSYGTNSGVAIYHRYKAGNYRS--PPITADIELALLRA                    219
batCOV5-M   MIKRQAYGTNSGVAIYHRYKAGNYRR--PPIIQDQELALLRA                    220
SARS2-M     LGASQRVAGDSGFAAYSRYRIGNYKLNTDHSSSSDNIALLVQ                    222
SARS-M      LGASQRVGTDSGFAAYNRYRIGNYKLNTDHAGSNDNIALLVQ                    221
            :   *  . :**.* * **: ***:         . ::***
```

Figure S2. Sequence alignment of several betacoronavirus M proteins by ClustalW(1, 2). TM helices are indicated by cylinders and labeled from TM1 to TM3. The batCOV5-M$_{CTD}$ sequence is highlighted in cyan. Basic residues selected from the “horizontal groove” and the “vertical groove” in this study, including R42, R131, K150, H155 and K172, are highlighted in magenta (highly conserved) and in green (less conserved). Asterisks (*) indicate identical residues. Colons (:) indicate strong similarities. Periods (.) indicate weak similarities. MERS-M, MERS-CoV M protein; batCOV5-M, *Pipistrellus* bat coronavirus HKU5 M protein; SARS2-M, SARS-CoV-2 M protein; SARS-M, SARS-CoV M protein.

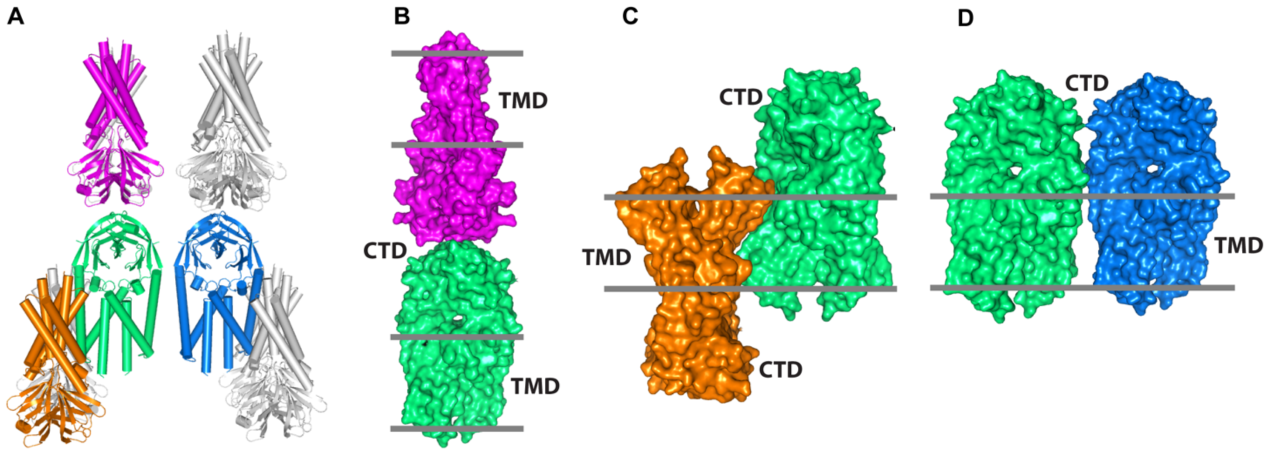

Figure S3. Inter-dimer contacts of batCOV5-M$_{xtal}$. (A) Crystal packing of the batCOV5-M$_{xtal}$ structure. Using the green dimer as a center, three different inter-dimer contacts are observed, and the contacting dimers are colored in magenta, orange and marine blue. (B) A "head-to-head" contact mediated via CTD. (C) An "opposite" packing mode mediated via TMD. (D) A "side-by-side" contact mediated via CTD.

```
MERS-N        MASPAAP------RAVSFADNNDITNTNLSRGRGRN----PKPRAAPNNTVSWYTGLTQH    50
batCOV5-N     MATPAPP------RAVVFANDNETPTNSQRSGRPR-----TKPRPAPNTTVSWFTGLTQH    49
SARS2-N       MSDNGPQ-NQRNAPRITFGGPSDSTGSNQNGERSGARSKQRRPQGLPNNTASWFTALTQH    59
SARS-N        MSDNGPQSNQRSAPRITFGGPTDSTDNNQNGGRNGARPKQRRPQGLPNNTASWFTALTQH    60
              *:  ..         : *.. .: . ..    *        :*:  **.*.**:*.****

MERS-N        GKVPLTFPPGQGVPLNANSTPAQNAGYWRRQDRKINTGNG-IKQLAPRWYFYYTGTGPEA    109
batCOV5-N     GKQPLAFPPGQGVPLNANSTPAQNAGYWRRQDRKINTGNG-TKPLAPRWYFYYTGTGPEA    108
SARS2-N       GKEDLKFPRGQGVPINTNSSPDDQIGYYRRATRRIRGGDGKMKDLSPRWYFYYLGTGPEA    119
SARS-N        GKEELRFPRGQGVPINTNSGPDDQIGYYRRATRRVRGGDGKMKELSPRWYFYYLGTGPEA    120
              **  * ** *****:*:** * :: **:**  *::. *:*  * *:******* ******

MERS-N        ALPFRAVKDGIVWVHEDGATDAP-STFGTRNPNNDSAIVTQFAPGTKLPKNFHIEGTGGN    168
batCOV5-N     NLPFRSVKDGIIWVHENGATDAP-SVFGTRNPANDPAIVTQFAPGTTLPKNFHIEGTGGN    167
SARS2-N       GLPYGANKDGIIWVATEGALNTPKDHIGTRNPANNAAIVLQLPQGTTLPKGFYAEGSRGG    179
SARS-N        SLPYGANKEGIVWVATEGALNTPKDHIGTRNPNNNAATVLQLPQGTTLPKGFYAEGSRGG    180
               **: : *:**:**  :** ::* . :***** *:.* * *:. **.***.*: **: *.

MERS-N        SQSSSRASSVSRNSSRSSSQGSRSGNSTRGTSPG--PSGIGAVGGD---LLYLDLLNRLQ    223
batCOV5-N     SQSSSRAS--SRSSSRSSSRNGRSNNSSRNASPA--PHGVGDVVGAGTLSVLLDLQKRLA    223
SARS2-N       SQASSRSS----SRSRNSSRNSTPG-SSRGTSPARMAGNGGDAALA---LLLLDRLNQLE    231
SARS-N        SQASSRSS----SRSRGNSRNSTPG-SSRGNSPARMASGGGETALA---LLLLDRLNQLE    232
              **:***:*    . **..*:.. .. *:*. **.  . . * .       : **  ::*

MERS-N        ALESGKVKQSQPKVITKKDAAAAKNKMRHKRTSTKSFNMVQAFGLRGPGDLQGNFGDLQL    283
batCOV5-N     DLEAGKGNK-QPKVITKKDAQAAKQKMRHKRVATKGYNVVQAFGMRGPGPLQSNFGDMQY    282
SARS2-N       SKMSGKGQQQQGQTVTKKSAAEASKKPRQKRTATKAYNVTQAFGRRGPEQTQGNFGDQEL    291
SARS-N        SKVSGKGQQQQGQTVTKKSAAEASKKPRQKRTATKQYNVTQAFGRRGPEQTQGNFGDQDL    292
                 :** :: * :.:***.*  *.:* *:**.:** :*:.**** ***   *.**** :

MERS-N        NKLGTEDPRWPQIAELAPTASAFMGMSQFKLTHQNNDDHGNPVYFLRYSGAIKLDPKNPN    343
batCOV5-N     NKLGTEDPRWPQIAELAPSASAFMSTSQFKVTHQSNDENGEPVYFLSYSGAIKLDPKNPN    342
SARS2-N       IRQGTDYKHWPQIAQFAPSASAFFGMSRIGMEVTPSG------TWLTYTGAIKLDDKDPN    345
SARS-N        IRQGTDYKHWPQIAQFAPSASAFFGMSRIGMEVTPSG------TWLTYHGAIKLDDKDPQ    346
               : **:  :*****::**:****:. *:: :    ..       :* * ****** *:*:

MERS-N        YNKWLELLEQNIDAYKTFPKKEKKQK-----APKEESTDQMSE----PPKEHR----VQG    390
batCOV5-N     YNKWMEILDANIDAYKSFPKKERKQKPSGDDAATAPATSQMEDVPELPPKQQRKKRVVQG    402
SARS2-N       FKDQVILLNKHIDAYKTFPPTEPKKD------KKKKADETQAL----PQRQKKQQTVTLL    395
SARS-N        FKDNVILLNKHIDAYKTFPPTEPKKD------KKKKTDEAQPL----PQRQKKQPTVTLL    396
              ::. : :*: :*****:** .* *:.       .  : .        * ::::    .

MERS-N        --TQRTRTRPSVQ-PGPMIDVNTD--                                      411
batCOV5-N     SIPQRSAGVPSFEDVVDAIFPDSEA-                                      427
SARS2-N       PAADLDDFSKQLQQSMS--SADSTQA                                      419
SARS-N        PAADMDDFSRQLQNSMSGASADSTQA                                      422
                .:      ..:        ::
```

Figure S4. Sequence alignment of several betacoronavirus N proteins by ClustalW(1, 2). N_1 is highlighted in cyan, N_2 in yellow and N_3 in green. Asterisks (*) indicate identical residues. Colons (:) indicate strong similarities. Periods (.) indicate weak similarities. MERS-N, MERS-CoV N protein; batCOV5-N, *Pipistrellus* bat coronavirus HKU5 N protein; SARS2-N, SARS-CoV-2 N protein; SARS-N, SARS-CoV N protein.

Supplementary references

1. Thompson JD, Higgins DG, & Gibson TJ (1994) CLUSTAL W: improving the sensitivity of progressive multiple sequence alignment through sequence weighting, position-specific gap penalties and weight matrix choice. *Nucleic Acids Res* 22(22):4673-4680.
2. Combet C, Blanchet C, Geourjon C, & Deleage G (2000) NPS@: network protein sequence analysis. *Trends Biochem Sci* 25(3):147-150.
